# Weight cycling and its effects on muscle mass, sarcopenia and sarcopenic obesity

**DOI:** 10.1007/s11154-025-09963-8

**Published:** 2025-04-15

**Authors:** Mauro Zamboni, Anna Giani, Francesco Fantin, Andrea P. Rossi, Gloria Mazzali, Elena Zoico

**Affiliations:** 1 Division of Geriatric Medicine, Department of Medicine, University of Verona, Piazzale Stefani 1, 37126 Verona, Italy; 2 Centre for Medical Sciences-CISMed, Department of Psychology and Cognitive Science, Section of Geriatric Medicine, University of Trento, Rovereto, Italy; 3 Division of Geriatrics, Department of Medicine, Ospedale Cà Foncello, 31100 Treviso, Italy

**Keywords:** Weight cycling, Muscle, Sarcopenia, Sarcopenic Obesity, Aging, Body composition

## Abstract

The prevalence of obesity is rising around the world, as the number of subjects dieting and experiencing weight loss followed by unintentional weight regain, the so-called weight cycling (WC). In this narrative review we sought to reveal the effects of WC on body composition as well as to evaluate if WC may represent a risk factor for sarcopenia and sarcopenic obesity. The relative changes in lean and fat compartments after WC have been shown to depend on several factors as the degree of energy deficit, the rate of weight loss and baseline body weight, as well as sex, age, physical activity and subject’s metabolic or hormonal status. Effects of WC on body compartments may be underestimated depending on body composition measurements, interference of physical exercise, number of WC, age and population characteristics. Studies using the most precise methods to assess body composition changes, as well those with higher number of WC and/or including old subjects, show that with WC, lower fat free mass (FFM) is regained with a mismatch between muscle and fat. In addition, when WC is involuntary in old age, it drives and accelerates the age-related loss of FFM, in particular in frail populations. Finally, an association between WC and sarcopenia or sarcopenia obesity, was also observed when evaluating strength together with WC-related body composition changes. In conclusion WC may act as an accelerator of biological aging, and it could be further investigated as a potential risk factor for sarcopenia or sarcopenic obesity.

## Introduction

Prevalence of overweight and obesity has increased in the last decade, with females being more likely to be affected by overweight and obesity than males [1, 2]. Contextually, even the number of subjects who are dieting has increased [3–5] and what is noteworthy is that it is not just people with excess weight who are dieters, but also normal weight subjects [5, 6]. However, a substantial proportion of dieters undergo weight cycling (WC), which is defined as intentional weight loss followed by unintentional weight regain. WC, with periodic decrease and increase in body weight, did not receive a unique characterization so that different operative definitions (concerning the number as well as the magnitude of weight cycles) have been used in different studies [7, 8].

One of the first studies describing the prevalence of WC was conducted in Finland with data obtained from the National FIN-RISK 1997 survey, in a wide representative sample of men and women, through the administration of a questionnaire investigating the number of voluntary weight loss and involuntary regain in a 10-year follow-up period [9]. In this cohort of 3320 men and 3540 women with an age range from 25 to 64 years, 7% of men were defined as severe and 11% as mild weight cyclers, whereas in women, 10% and 19% were classified as severe and mild weight cyclers respectively [9].

Data from the 1999–2002 National Health and Nutrition Examination Survey (NHANES), examining a subsample of 1310 adults aged 20–84 years with overweight or obesity, and a substantial weight loss, show, after 1 year, in the 33.5% of them, a regain > 5% of weight [10]. Even though in this study it was not possible to examine if the weight loss was voluntary, subjects attempting to control their weight had a 1.8 times increased risk of weight regain [10].

Longitudinal studies show that at least one-third of dieters regain more weight than they lost, and in prospective studies adults who were on a diet during childhood and adolescence, present more pronounced weight gain and obesity [11].

Among individuals attempting to lose weight, WC has been found to be more prevalent in women and in athletes who need to meet specific weight categories [7, 8]. Interestingly, WC is present also in normal weight subjects [12, 13].

Several observations seem to show that WC is associated with future weight gain more in normal weight than in subjects with obesity [12–14]. Moreover, it has been found that WC is associated with increased risk of Type 2 Diabetes [15], poor cardiometabolic outcomes [12, 16, 17] and increased cardiovascular mortality [16, 17], independently of obesity [18]. Association between WC and increased risk of eating disorders has been also observed [19]. The effects of WC on body composition changes have been the object of several studies in the past, with discrepant results.

The purpose of the present narrative review was to analyze published articles on the effects of WC on body composition over a wide age range and to see if WC may represent a risk factor for sarcopenia or sarcopenic obesity.

### Effect of weight change on body composition: background assumptions

Changes in body weight, negative or positive, involve fat free mass (FFM) and fat mass (FM) [20–25]. However, the relative contribution of FFM and FM changes after weight loss has been historically shown to depend on:

1 degree of energy deficit and thus rate of weight loss: with a daily calorie deficit of 1000 kcal/day, the rate of weight loss per day has been shown to be approximately 0.3 pound per day and the composition of weight lost is considered acceptable, in line with the general consensus, when composed of 75% of FM and 25% of FFM. When the daily energy deficit increases, the rate of weight loss increases too, but the quality of weight lost declines: the larger the energy deficit the greater the ∆ FFM/∆ Weight [20]. As a consequence, if great energy deficit goes on by the time, a depletion of FFM could be expected.

2 initial body weight/adiposity: for individuals with a 1000 kcal per day of deficit, ∆FFM/∆Weight is higher in normal weight individuals than in subjects with obesity and declines steeply as body fat increases [21].

Interestingly, in the Minnesota Starvation Experiment it was observed that the lean-fat partitioning characteristic of the individual during weight loss was conserved even during weight regain, and that initial body fat % was the strongest predictor of interindividual variability in lean-fat partitioning. In men the leaner the subjects, the greater the fat gained after refeeding: in fact, after nutritional rehabilitation following a body weight loss of more than 25% of the baseline body weight, a mean fat overshooting of 4 kg was found (ranging from 0 to 9 kg) and fat regain was greater in subjects with lowest BMI values, with an inverse relationship between the kg of fat regain and the initial body fat % [13, 23].

If we consider the combined presence of points 1 and 2, a high degree of energy deficit erodes lean body mass much more than expected in normal weight subjects than in individuals with excess weight.

Actually, the proportion of weight loss as FFM over time has been shown to be also influenced by other factors such as sex, age, presence of inactivity, type and amount of physical activity, subject’s metabolic state or hormonal response [22] as well by the fact that ΔFFM/ΔWeight is larger at the beginning of dieting and then gradually declines as dieting proceeds [26].

### Physiological mechanisms of weight-cycling

Several mechanisms have been proposed for explaining WC. The most relevant seem to be:

- Protein-fat partitioning

The energy partitioning between protein and fat during weight regain could be driven by the memory of the initial body composition, in particular initial body fat % (initial ratio of fat-to-FFM) [27].

- Thermogenesis

Thermogenesis is downregulated during weight loss and may persist suppressed during weight recovery by a magnitude that has been shown to be proportional to the state of depletion of the fat stores [28] as well as by the fraction of the body’s protein mobilized as an energy reserve, as important as the initial body fat % [27].

Further, it has also been hypothesized a “nonspecific” control of thermogenesis, that likely occurs primarily in organs/tissues with a high specific metabolic rate. This system is primarily under control of the sympathetic nervous system, whose functional state is regulated by the interacting signals deriving from a variety of environmental stresses, including food deprivation, deficiency of essential nutrients, excess energy intake, and exposure to cold or to infections [27].

- Adipocytes metabolic memory

In models of diet-induced obesity and weight loss to study the transcriptional memory of obesity, it has been found that the ‘metabolic memory’ of obesity may be localized in adipocytes, in particular in genes that play a role in food intake regulation and which expression in obese mouse and human adipocytes persists after weight loss [29].

These findings have been confirmed recently because it has been found that cellular transcriptional changes, evaluated by the single-nucleus RNA sequencing of mouse and human adipocytes, persist after weight loss suggesting the existence of an “obesogenic memory”, consisting in obesity-induced alterations in the epigenome of adipocytes that accelerate weight gain [30].

## Methods

In this narrative review we sought to show the effects of WC on body composition in patients with a wide range of age and body weight. Moreover, we would evaluate if WC may represent a risk factor for sarcopenia or sarcopenic obesity. Both voluntary and involuntary weight loss followed by weight regain in the elderly were included.

A bibliographic preliminary search was performed through the main medical search engines, using the following main keywords: weight cycling or yo-yo diet and weight loss, weight regain, body composition, FM, body fat percentage (FM%), FFM or lean body mass (LBM) as reported by the authors. This search was followed by a manual, in-depth study of articles and their respective references. We decided only to include paper in English, published between 1989–2024, describing studies that used validated body composition methods (i.e. excluding those using skinfold thickness measurements), with data about FM and/or FFM. We did not included studies conducted on athletes, since they are not generalizable to the whole population and that with weight loss induced through the use of drugs or bariatric surgery.

After selecting articles based on their relevance to the research questions, 26 papers were finally included in the critical synthesis and revision of the literature (Tables 1 and 2) [31–56].

**Table 1:** Main characteristics of studies reporting weight cycling effects on body composition after voluntary weight loss and regain

Author, year of publication	Study sample (n, F/M, age, BMI)	Weight cycling (number, definition)	Type of study and intervention (diet ± physical activity)	Method of body composition evaluation	Effects on Fat	Effects on Muscle	Results
*Van Dale D, 1989, *[31]	23 women with obesity (age range 20-45y; mean BMI 33.5 kg/m^2^)	** > WC**: at least twice lost and regained > = 10 kg in the last 5 y (self-reported) to define WC and NWC women	**Intervention study**: VLCD (1–5 wks 722 kcal formula diet; 6–14 wks 528 kcal formula diet + 312 kcal of normal food + 2 wks of 722 kcal formula diet) + exercise (aerobic 3 h/wk and strength 1 h/wk)	Underwater weighting	Same FM between WC and NWC subjects. Same FM reduction between WC and NWC subjects (differences only in relation to exercise)	Same FFM between WC and NWC subjects. Trend toward > FFM reduction in WC women	** = FM = ↓FFM**
*Manore MM, 1991, *[32]	11 normal weight WC women and 12 controls (mean age 23y; mean BMI 23.6 kg/m^2^)	** > WC:** subjects having dieted (−1000 kcal/d below REE) for 7-10d, 4 times during the past 3 years (self-reported)	**Observational retrospective study**	Underwater weighting	FM% and BMI were significantly higher in cyclical dieters compared to controls	FFM similar between dieters and controls	**↑ FM% = FFM**
*Jebb SA,* *1991, *[33]	11 women with obesity (age range 35-55y) (mean BMI 31.4 kg/m^2^)	** > WC:** defined as patients with previous history of weight loss > = 6 kg on 2 or more occasions	**Intervention study**: 18 wks (2 wks of VLCD: 445 kcal/die, 4 wks ad libitum, for 3 times) no recommendation or food record in free-living periods. No advice for exercise	Different body composition methods	Weight loss as FM between 105 and 67% of the weight lost in WC women	Weight loss as FFM between −5 to 33% of the weight lost in WC women	** = FM = FFM**
*Wadden TA, 1992, *[34]	50 adult women with obesity (mean age 40.1y, mean BMI 37.9 kg/m^2^)	** > WC** defined by the “Weight and Lifestyle Questionnaire”: record of all diets with weight loss > = 4.5 kg	**Intervention study:** different 6-mths diets (0–2 wks 1200 kcal diet; 2–13 wks randomization into VLCD of 420, 660, 800 kcal/die with formula products; 14–19 wks deficit diet of 1000 kcal with food reintroduction; 19–24 wks 1000–1500 kcal balance diet). No advice for exercise	Underwater weighting	Association of WC with increased FM and waist and hip circumference at baseline. No association of WC with changes in FM	No association of WC with changes in FFM	**↑ = FM ↑waist, ↑hip= FFM**
*Van der Kooy K, 1993, *[35]	15 women with obesity, 17 obese men (age range 25-51y and BMI range 28–38 kg/m^2^)	**1 WC** defined as regain > 30% of the weight loss after intervention (mean FU 67 wks)	**Intervention study:** diet with energy deficit of 4.2 MJ/day for 13 weeks followed by no other diets until the re-evaluation on average at 67 wks. No advice for exercise	DXA and MRI (for fat distribution)	FM%, waist circumference, WHR and VAT area after weight regain increased but were below initial values; SAT area slightly increased the basal value	No data	** = FM** **↓FM%** **↓ WHR** **↓VAT/SAT**
*Wadden TA, 1996, *[36]	12 women with obesity (mean age 38.8 y; mean BMI 36.7 kg/m^2^)	**1 WC** defined as regain within +—5 kg of their initial weight after a weight reduction program (FU 2-3y)	**Intervention study:** 1 wk 1.200 kcal/d of balanced diet; 2–12 or 16 wks VLCD of 420–800 kcal/d; > 16 wks reintroduction of normal food. No advice for exercise	Underwater weighting	Failed to show that WC increased the deposition of fat in the upper body or the ratio of fat to lean tissue	WC showed no association with changes in FFM	** = FM** ** = WHR** ** = FM/FFM** ** = FFM**
*Zamboni M, 1996, *[37]	11 pre- and 9 post-menopausal women (mean age 31.7y and 54.3y; mean BMI 34.8 and 38.9 kg/m^2^ respectively)	**1 WC:** regain of initial body weight after a nutritional intervention (FU 11.1 ± 6 mths)	**Intervention study:** 2 weeks of VLED of 400 kcal/d followed by low energy diet of 1200 kcal/d. No advice for exercise	CT	No increase in total abdominal adipose tissue and VAT at regain of weight, with no difference between pre- and post-menopausal women	No data	** = total abdominal FM** ** = VAT**
*Hammer RL, 1998, *[38]	44 pre-menopausal women with obesity	**1 WC** defined as subjects who failed to maintain their weight loss at 6 and 12 mths of FU after intervention	**Intervention study**: 12 or 16 wks of low-fat diet of 1500 kcal ± supervised aerobic exercise 30 min for 4–5 day/wk	Underwater weighting	Subjects regaining > of 75% of initial weight (n = 19) had no increase in FM% or WHR above pretreatment levels	No data	** = FM** ** = WHR**
*Kajioka T, 2002, *[39]	5 young normal weight women (mean age, 24.6 y; mean BMI 20.5 kg/m^2^)	**2 WC** in patients with no history of WC	**Intervention study**: 0–30 d low calorie diet (< 1200 kcal/day); 30–44 d free living; 44–74 d low calorie diet (1200 kcal/day) 74–180 days free living. No advice for exercise	Air Displacement Plethysmography	After 2 WC, FM and WHR were not significantly changed	After 1 WC, LBM was unchanged; after 2 WC LBM was significantly decreased compared to baseline	** = FM** ** = WHR** **↓LBM**
*Byrne NM, 2003, *[40]	18 white, 22 black young pre-menopausal women with overweight (age range 20–46 y; BMI range 27–30 kg/m^2^)	**1 WC**: defined as losing > 10 kg or reaching BMI < 25 kg/m^2^ (1y FU: evaluations at baseline, after weight loss and after weight regain)	**Intervention study:** VLCD 800 kcal/die until lost > 10 kg or BMI < 25 kg/m^2^ followed by maintenance diet. No advice for exercise	DXA	Body weight, FM and FM% with weight regain increased significantly but not to basal values (no racial difference)	LBM with weight regain did not return to basal value; significant increases in limb LBM but trunk LBM remained lower	** = FM** **↓total LBM** **↓Trunk LBM** ** = limb LBM**
*Graci S, 2004, *[41]	459 patients with obesity, 340 F, 119 M (age 19–65 y, mean 43.6y; BMI 30–69 kg/m^2^, mean 41.0 kg/m^2^)	** > WC**: defined by the “Weight and Lifestyle Questionnaire”: record of all diets with weight loss > = 4.5 kg	**Observational, Retrospective study**	BIA	Positive correlation between WC and FM% and waist in both genders; these correlations disappeared after adjustment for age and BMI in women	No data	**↑ FM%** **↑ waist** **No more after adjustment for age and BMI**
*Strychar I, 2009, *[42]	121 post-menopausal women with obesity (mean age 58y; mean BMI 32 kg/m^2^)	** > WC** defined as non-dieting (0), low (1 time), moderate (2–3 times), and frequent (> 4 times) dieters based on the frequency of going on a diet and loosing > 10 kg	**Interventional study**: 6-month randomized weight-loss program with diet ± resistance training	DXA	Frequent cyclers had higher BMI, higher FM%, larger waist circumference, than non-cyclers	Frequent cyclers had borderline lower LBM% than non-cyclers	**↑ FM** **↑ waist** **trend ↓LBM**
*Anastasiou CA, 2010, *[43]	32 young normal weight healthy women (mean age 21.1 y, mean BMI 21.2 kg/m^2^)	** > WC:** defined according to “25-item Weight Cycling Questionnaire” (self-reported)	**Observational, retrospective study**	DXA	Repeated attempts to lose small amounts of weight are associated with ↑ FM	Not evaluated	**↑ FM**
*Yoo HJ, 2010, *[44]	109 Korean patients with overweight and obesity (F/M) (mean age 41.4 or 44.9y, mean BMI 27.7 or 27.0 kg/m^2^ in WC and NWC group)	** > WC:** defined as a body weight change > 5% of the initial body weight in the previous 2y WC group (*n* = 47) NWC group (*n* = 62)	**Intervention study:** 12-weeks of Protein-Rich Oriental Diet Program + physical exercise (aerobic exercise > 4 days/w and strength training twice a week)	BIA	After intervention less reduction in FM% in WC subjects, also after adjustment for age	After intervention more reduction in FFM, also after adjustment for age	**↑ FM** **↓ FFM**
*Beavers KM, 2011, *[45]	78 post-menopausal women (age 50-70y; mean BMI 33.3 kg/m^2^)	**1 WC**: defined as weight regain > = 2 kg during the FU period, after a 5-mths intervention (FU of 6 and 12 mths)	Intervention study: 1–5 mths randomized controlled trial with diet (calorie restriction of 400 kcal/die) ± moderate-intensity or vigorous-intensity aerobic exercise (3 days/wk); 6–12 mths counseling for maintenance of WL	DXA	Average FM was higher at 6 and 12 mths FU than at the postintervention; absolute FM was lower than at baseline. WHR decreased significantly by 12 mths of FU	LM did not significantly change during the FU period and, at 12-mths was still lower than baseline Decreasing trend in LM/FM	**↓ FM** **↓ WHR** **↓ LM** **↓ LM/FM**
*Bosy-Westphal A, 2013 *[46]	47 healthy men and women with overweight and obesity (age range 22–45 y; BMI > 29 kg/m^2^)	**1 WC**: weight regainers (> 30% of their weight loss, n 27) and weight-stable (< = 20% of their weight loss, n 20); FU 6 mths	**Intervention study:** 13-wks low-calorie balanced diet (diet containing 800–1000 kcal day where 434 kcal were supplied as a VLED). No advice for exercise	DXA Air-displacement plethysmography MRI	No differences in weight loss or loss of FM between groups. Higher regain in AT of the extremities in F (gluteofemoral pattern)	No differences in loss of FFM between groups. Higher regain of muscle at the extremities than at the trunk	** = FM** ** = WHR (gluteofemoral pattern for F)** ** = FFM**
*Pownall HJ, 2015, *[47]	1019 M/F subjects with overweight or obesity and diabetes (age range 45-76y) (BMI > 25 kg/m^2^) (3 ethnical groups) “Look AHEAD Study”	**1 WC:** defined as regain of weight in the following 8 y, after 1-year program designed for a weight loss of > = 7%; FU at 1y and 8 y	**Intervention study:** randomized trial with diet (1200–1500 kcal/day) or diet (1500–1800 kcal day) + physical exercise (175 min/wk) vs general information about healthy diet and physical activity	DXA	At 1 year, the intervention group lost fat but between years 1 and 8 regained fat (100%)	At 1 year, the intervention group lost lean mass, but despite regaining weight, lost lean mass between years 1 and 8	** = FM** **↓ LBM**
*Fothergill E, 2016, *[48]	14 adult men and women with obesity (6 M, 8 F) (mean age 34.5 y; mean BMI 49.5 kg/m^2^)	Study of **long-term effects of a single WC** (after 6 years) subjects who participated in “The Biggest Loser” 30-week weight loss competition”	**Observational study** The Biggest 30 wk loser competition: calorie-restricted diet greater than 70% of baseline energy requirements (30 wk) + 90 min/d (6 d/wk) of supervised vigorous aerobic training	DXA	After 6 years, most subjects regained weight; mean FM significantly increased in the 6y control but was below baseline	Mean FFM significantly increased in the 6y control but was below baseline	**↓ FM** **↓FFM**
*Chmelo EA, 2016, *[49]	12 F, 12 M elderly patients with overweight and obesity (age range 65–79 years; mean BMI 29.9 kg/m^2^)	**1 WC:** defined as regain some body mass from 5 to FU 23 mths	**Intervention study:** 5 mths trial of caloric restriction (−600 kcal) + resistance training (3 days/wk) (18 mths of FU)	DXA Thigh by CT	Total FM and all thigh fat volumes increased during the post intervention but not to the basal level	Thigh muscle volume decreased and intermuscular fat increased	** = FM** **↓ FFM** **↓ limb LBM↑ IMAT **
*Dandanell S,* *2017, *[50]	2120 F/M participants (mean age 36y; BMI 40 ± 8 kg/m^2^)	** > WC:** from 1 to 4 intensive lifestyle interventions (ILIs) (n = 482 > 2 ILIs)	**Intervention study**: each ILI: hypocaloric diet (− 500 to − 700 kcal/day below energy expenditure) + daily exercise (1–3 h of mixed sports activities) for 11–12 wks	BIA	The ratio between loss of FM and FFM decreased with the number of interventions	The ratio between loss of FM and FFM decreased with the number of interventions	**↑ FM** **↓ FFM**
*El Ghoch M, 2018, *[51]	38 adult men and women with obesity (14 M, 24 F) (mean age 54.7y; mean BMI 43.5 kg/m^2^)	**1 WC:** significant weight loss (> = 5%) followed by subsequent weight regain > = 30% of the weight lost. Mean FU 20.3 mths)	**Intervention study:** residential program for severe obesity of 21 d with balanced low-calorie diet (1200 kcal/day) + daily aerobic activity and group cognitive behavioral treatment	BIA	No change in waist circumference and FM%	No change in FFM	** = FM%** ** = waist circumference** ** = FFM**
*Rossi AP, 2019, *[52]	60 M, 147 F adult subjects with obesity (mean age 52.6y, mean BMI 37.9 kg/m^2^)	** > WC:** defined as a voluntary weight loss ≥ 3 kg followed by an involuntary weight regain ≥ 3 kg**.** Severe weight cyclers (6 or > WC)	**Observational, retrospective study**	DXA	No data available	Severe weight cyclers showed a 3.8-times increased risk of low muscle mass, also after adjustement for age, sex and FM	**↓ FFM**
*48. Tannir H, 2020, *[53]	129 adult women with obesity (mean age 38y; mean BMI 35.7 kg/m^2^)	** > WC**: defined as at least 2 cycles of WL ≥ 3 kg, followed by involuntary weight regain of ≥ 3 kg	**Observational study:** comparison between WC and non-WC obese women	BIA	WC women had a higher FM, and trunk fat mass than non-WC after adjustment for age and FFM	WC women had lower %FFM than non-WC	**↑ FM** **↑ trunk FM** **↓FFM%**

*BIA* = bio-impedance analysis; *BMI* = Body mass index; *CT* = computed tomography; *d* = days; *DXA* = Dual Energy X-ray Absorptiometry; *F* = female; *FFM* = fat free mass; *FM* = fat mass; *FU* = follow-up; IMAT = intermuscular adipose tissue; *LBM* = lean body mass; *M* = male; *MRI* = magnetic resonance imaging; mths = months; *NWC* = non weight cycling; *VLED* = very low-energy diet; *VLCD* = very low-calorie diet; *WC* = weight cycling; *WHR* = waist to hip ratio; *wks* = weeks; *WL* = weight loss; *y* = years. [ ] in squared brackets the articles are numbered as in the reference section. The inclusion of physical activity in the intervention program of the different studies was underlined in the table text

**Table 2:** Main characteristics of studies reporting the effects of involuntary weight loss and regain on body composition

Author, year of publication	Study sample (n, F/M, age, BMI)	Weight changes (definitions)	Type of study and intervention (diet ± physical activity)	Method of body composition evaluation	Effects on Fat	Effects on Muscle	Results
*Lee JS, 2010, *[54]	147 elderly patients with weight changes (F/M) and 147 weight stable age, gender and race matched controls from the Health ABC Study (mean age 73y, mean BMI 27 kg/m^2^)	Weight loss of 3% or > (1st y FU) with regain of within ± 3% (2nd y FU) (comparison of patients with stable weight and with weight changes)	**Longitudinal observational study** 2y FU	DXA	% increase in FM was greater than % loss in LBM in patients with weight changes (F/M);	Men with weight changes had a significantly lower absolute LBM and LBM/total mass compared to controls	**↑FM** **↓LBM ↓LBM/total mass (in men with weight changes)**
*Murphy RA, 2014, *[55]	1.975 participants (1.044 women, 931 men, 37% black) from the Health ABC Study	Weight change of 5% (gain and loss) from year to year or from 1 to 6 years of FU (comparison of patients with stable, weight loss or gain and with weight loss and regain)	**Longitudinal observational study** (5 y of FU for body composition and 8 y of FU for clinical outcomes)	DXA	Patients with weight loss and regain (F/M) had the greatest % CV of FM increase compared to other groups	Patients with weight loss and regain (F/M) had the greatest % CV of LBM decrease compared to other groups	**↑FM %CV, ↓LBM %CV (in patients with weight loss and regain)**
*Yates T, 2024, *[56]	622 F/M participants from the Walking Away from Type 2 Diabetes trial (F = 35.4%) (mean age 63.6 y; mean BMI 32.0 kg/m^2^)	Weight loss of ≥ 5% between baseline and 12 mths, regained between 12 and 24 mths (comparison between seven different weight loss and weight gain trajectories)	**Longitudinal observational study** (2 y of FU)	BIA	Patients with weight loss and regain regained FM to baseline levels (adjusted for confounders)	Patients with weight loss and regain lost FFM (adjusted for confounders)	** = FM** **↓ FFM**

*BIA* = bio-impedance analysis; *BMI* = Body mass index; CV = coefficient of variation; *DXA* = Dual Energy X-ray Absorptiometry; *F* = female; *FFM* = fat free mass; *FM* = fat mass; *FU* = follow-up; *LBM* = lean body mass; *M* = male; *mths* = months; *wks* = weeks; *y* = years

### Weight cycling (WC) and body composition: an analysis of the literature

A large number of papers analyzed body composition changes in weight cyclers and had conflicting results. Differences in study characteristics may, in part, explain the discrepant results of the literature.

Tables 1 and 2 summarize the main results of the articles selected through database searches and present them by publication date [31–56]. Studies with voluntary [31–53] and involuntary [54–56] weight loss and regain are separately presented in Tables 1 and 2 respectively. The main characteristics of the study sample, WC definition, study design and type of intervention as well as methods of body composition used, were summarized in the two tables to facilitate the comparison between different studies and the interpretation of the results.

### Voluntary weight loss and weight cycling: 1 cycle of weight loss and regain

Most of the papers that present data on the effects of WC on body composition were conducted in in patients with overweight and obesity [31, 33–38, 40–42, 44–53], involved in weight control programs, and only a few in normal weight subjects [32, 39, 43].

The majority are represented by intervention studies, where subjects with overweight and obesity, undergoing different nutritional treatments, associated or not with physical exercise, were characterized by body composition changes at the end of the intervention and after weight regain with very variable lengths of follow-up, from a few months to many years (Table 1). These studies generally provide data about body composition changes after a single weight loss cycle [35–38, 40, 45–49, 51] (Table 1).

In a small group of 12 young women affected by obesity, Wadden et al. [36] did not find any significant increase in either FM% or FM when body weight returned to baseline 2–3 years after a weight loss program with very low-calorie diet (VLCD). Moreover, in the same study, WC showed no association with FFM changes, which led the authors to conclude that their results did not support the clinical hypothesis that WC may adversely affect body composition [36]. Similarly, in a wider study sample of 44 premenopausal women with obesity, Hammer et al. [38], reached the same conclusion, confirming no effect on total body FM after failure to maintain the weight loss at 6 and 12 months. The stability of FM and FFM, upon regaining of body weight after a nutritional program, was further confirmed in a sample of healthy young subjects of both sexes with excess weight [46], as well as in different racial groups [40].

Obesity studies often assess women only, presented small sample size with a potential lack of statistical power and do not take into account potential confounders as physical activity [36, 38, 40, 46]. In fact, even though they used accurate methods for evaluating body composition, data about physical activity are often missing and only one study [38] provided a physical exercise program to dieters, with only endurance but no strength training.

It has been well established that one of the main factors influencing body composition is age [57–60]. WC has been indicated as a hypothetical factor that magnifies age-related changes in body composition [57, 60, 61]. Thus, effects of WC on body composition may be different in subjects of different age groups, with more relevant body composition changes in older subjects [45, 47, 49], compared to young adults [36, 38, 40, 46] or middle-aged subjects [35, 51].

Only a few papers have included adults with an age range of 40 to 60 years [35, 51]. These studies were conducted in small samples of patients with a wide range of BMI and conclude for a substantial stability of body composition, and in particular of FM, after a single WC [35, 51].

In a 5-month nutritional trial conducted on 78 women affected by obesity aged 50–70 years old, after significant weight reduction (13%) those who regained weight after 1-year of follow-up had a reduced lean-to fat mass ratio: for every 1 kg of fat lost during the weight-loss intervention, 0.26 kg of lean tissue was lost and for every 1 kg of fat regained over the following year, only 0.12 kg of lean tissue was regained [45]. This study introduced the important concept that lean mass lost during weight loss in the elderly may be not fully recovered during weight regain, increasing the risk of sarcopenia and, in particular, sarcopenic obesity in old women [45].

These findings were confirmed in a sub analysis of the Look-AHEAD Study in 1,019 elderly men and women with diabetes and BMI ≥ 25 kg/m^2^, participating to the control or to the intervention group, consisting of a 1-year program of low-calorie diet with or without physical activity [47]. At 1 year, the intervention group lost significantly more fat and lean mass compared to the controls; however, despite regaining weight and fat mass during the 8-year follow-up, elderly subjects of the intervention group lost lean mass with values approaching those of the control group [47].

All these data were further confirmed in a small sample of elderly subjects with BMI between 27 and 35 kg/m^2^, where body composition changes were also evaluated by CT scan at the thigh level [49]. In this study, during a follow-up period of 18 months, weight loss and regain were associated with a regain in fat rather than in muscle, also at the thigh level, despite the fact that their calorie restriction program was associated with strength training [49].

The changes in body fat distribution after WC have been investigated only in a few publications [35–38, 45, 46] (Table 1). In particular, in the majority of studies, after 1 weight cycle, body fat distribution, anthropometrically evaluated through waist to hip ratio (WHR), was unchanged in both adult men [46] and women with obesity [36, 38, 46]. Moreover, the stability of body fat distribution after WC, was also confirmed by CT [37] and MRI measurements [35] respectively, in a small group of pre- and post-menopausal women with obesity [37] or in men and women with excess weight [35]; in these studies, no significant change in VAT emerged after weight loss and regain [35, 37]. Furthermore, in the study by Bosy-Westphal et al. [46], regain in adipose tissue of the extremities was disproportionally higher in women and lower in men, suggesting that WC may lead to a more gynoid pattern of body fat distribution, with an increased capacity for fat storage in women. This tendency toward an increased peripheral body fat redistribution with WC was confirmed in a trial conducted on a group of elderly post-menopausal women, where WHR was significantly reduced after 5 months of nutritional treatment followed by weight regain at 1-year follow-up [45]. It is possible that 1 WC cannot significantly impact body fat distribution hypothesizing that only multiple WC can determine central fat redistribution.

### Voluntary weight loss and weight cycling: more cycles of weight loss and regain

In a small group of young normal weight women, Kajioka et al. [39], tried to recreate the effects of WC on body composition, inducing 2 weight cycles with low-calorie diet not associated with physical exercise programs. Following the first weight reduction and regain phase, LBM recovered to baseline values, but interestingly, after the second weight cycle, LBM was significantly decreased compared to baseline [39]; FM and WHR were not significantly different after the 2 WC when compared to baseline values in the same group [39]. In other studies [32, 43] that recruited small groups of young normal weight women, FM significantly increased in women reporting a story of frequent WC compared to controls, whereas FFM was unchanged [32] or not evaluated [43]. Globally, results of these studies [32, 39, 43] with normal weight young subjects are difficult to generalize and interpret; firstly, because they were conducted only on women; secondly, because of the small sample size; thirdly, because of the self-reported nature of WC [32, 43] and the lack of data considering the effects of physical activity [32, 43] (Table 1).

A few studies were specifically designed to examine the effects of multiple cycles of weight loss and regain on body composition in adult subjects with overweight and obesity [31, 33, 50, 53] (Table 1). In two intervention studies published in the 1990s [31, 33], the effects of VLCD on weight reduction and body composition changes were evaluated in small groups of young adult women with obesity experiencing multiple weight cycles prior to the inclusion in the study. In particular, no significant changes in body composition were observed after 3 consecutive cycles of body weight loss and regain of small extent in 11 women with BMI > 30 kg/m^2^ [33]. In another study, women affected by obesity that reported at least two periods of significant weight loss and regain in the previous 5 years, after a program of VLCD associated to exercise, presented the same amount of FM loss and a trend toward a higher FFM loss compared to controls without previous WC [31].

Studies including larger samples of women with obesity also took into account age, but came to different and discrepant conclusions [50, 53]. In the observational study of Tannir et al. [53], conducted on 129 adult women with BMI > 30 kg/m^2^, participants with more WC presented a significant higher FM, and trunk FM than non-weight cycling subjects, after adjustment for age and FFM; moreover, weight cycling women had a significant lower FFM% than non-weight cycling subjects [53].

Similar evidence that multiple WC may be responsible for unfavorable body composition changes derives from the intervention study of Dandanell S. et al. [50]. In 482 men and women with obesity and at least two weight cycles through repeated intensive lifestyle interventions (hypocaloric diet and physical exercise), the ratio between loss of FM and loss of FFM decreased with the number of WC [50]; that is, the rates at which FM was lost decreased with the number of interventions, whereas the relative loss of FFM increased, indicating a shift towards an unhealthy body composition with expansion of fat and decrease of muscle compartment [50].

This unhealthy pattern of changes in body composition after repeated WC, was further confirmed in other studies, conducted also in middle aged and older subjects with obesity [34, 41, 42, 44, 52]. In a study by Wadden TA et al. [34], previous WC, as evaluated through the Weight and Lifestyle Questionnaire, was associated with an increase in FM and waist and hip circumferences at the beginning of the intervention. In a group of post-menopausal women with obesity participating in a 6-month weight-loss program, subjects who were frequent cyclers, had higher values of BMI, FM% and larger waist circumference as well as borderline lower LBM% at the beginning of the program, compared to non-cycler post-menopausal women [42]. Similar results emerged also in another retrospective study, conducted on 459 patients with a wide range of age and BMI [41]. However, in this study, the positive correlation between WC and FM% disappeared after adjustment for age and BMI [41].

The adverse effect on muscle compartment of repeated cycles of weight loss and regain appears in an intervention study in 109 Korean patients with overweight and obesity, after 12 weeks of Protein-Rich Oriental Diet Program, associated with physical aerobic and strength exercise [44]. After the intervention, subjects with WC history experienced less reduction in FM% and less preservation of FFM compared to the control group [44]. Furthermore, in a group of adult subjects with obesity, it was shown that severe weight cyclers presented a 3.8-times increased risk of low muscle mass, also after adjustment for age, sex and FM compared to non-weight cycler subjects [52].

### Involuntary weight loss and regain

A few longitudinal population-based studies analyzed the changes in weight and body composition trajectories after involuntary weight loss followed by weight regain, in wide samples of subjects from the general population who were free of serious disease and without disability [54–56] (Table 2). Older adults from the Health ABC Study, experiencing 3% of weight loss and regain over a 2-year period, had an overall greater loss of lean mass compared with those who maintained weight, independently from age, gender and race, which suggests a failure to conserve lean mass in old age in subjects experiencing involuntary weight loss and regain [54].

Similarly, in a bigger sample of participants to the Health ABC Study, with a longer follow-up of 5 years, weight stable and elderly subjects with involuntary weight loss and regain had similar LBM and FM at the end of the follow-up period, but patients with involuntary weight loss and regain showed the greatest % CV of FM increase, as well as the greatest % CV of LBM decrease, compared to the other groups [55]. However, in this study different weight trajectories were simultaneously compared over a 5-year follow-up, and participants with involuntary weight loss and regain were represented by the 19% and 14% of women and men respectively, with a scarce generalizability to the whole population due also to the selection of a well-functioning and healthy population of elderly [55].

Another population study of more than six hundred elderly subjects with overweight and obesity, participating to the Walking Away from Type 2 Diabetes trial, recruiting adults at-risk of type 2 diabetes from primary care, provided the opportunity to characterize weight trajectories during a 2-year period of follow-up. [56]. In fact, in this study no nutritional intervention was provided to patients that participated only to a behavioral intervention aimed at increasing physical activity through walking [56]. After adjustment for confounders, subjects who experienced involuntary weight loss and regain (defined as weight loss of ≥ 5% between baseline and 12 months, regained between 12 and 24 months) at the end of the follow-up lost 1.50 kg in FFM with no change in FM, even after adjustment for potential confounders [56].

This suggests that involuntary weight loss and regain may lead to a progressive loss in FFM at even relatively modest amounts of weight loss and regain in at-risk populations with overweight and obesity [54, 56]. This net loss of FFM may represent an acceleration of the natural loss of FFM observed even in weight stable adults with ageing [57–62], suggesting that involuntary weight loss and regain may act as an accelerator of biological aging, and that it could be a risk factor for sarcopenia and sarcopenic obesity.

### Weight cycling, sarcopenia and sarcopenic obesity

WC may be a new and relatively unexplored driver of sarcopenia and sarcopenic obesity [63]. To date, only a few articles have investigated the relation between weight changes and sarcopenia as a main object of study [52, 55].

Depending on the design and on the characteristics of the subjects enrolled, in some but not all studies, WC determines, both in subjects with normal weight [39] and in individuals with overweight or obesity [40, 42, 44, 45, 47–50, 52, 53], a miss-match between muscle and fat, with less regain in FFM, or concurrent fat gain and FFM loss, which makes the fat overshooting theory possible (mostly in normal weight subjects).

In the last decades, a wide consensus has led to a definition of sarcopenia and sarcopenic obesity which is not just based on the amount of muscle mass and fat but that also includes strength and/or physical performance [64–66]. As far as we know, only one study [52] has directly explored the relationship between sarcopenia and WC. Rossi et al. [52], defined sarcopenia, according to the FNIH (Foundation for the National Institutes of Health) criteria, as values of appendicular skeletal muscle mass (ASM)/BMI lower than those of a normal young reference population, whereas muscle function was evaluated by the use of handgrip strength. In a group of 60 males and 147 females with BMI > 30 kg/m^2^ participating in an inpatient nutritional and rehabilitative treatment, WC was retrospectively evaluated and defined subjects as non-weight cyclers (0–1 weight cycle), mild weight cyclers (2–5 weight cycles), or severe weight cyclers (6 or more weight cycles) [52]. The prevalence of sarcopenia was 50% and five times higher in participants with severe WC compared with those without [52]. Patients with obesity and severe WC presented lower muscle mass (ASM/BMI) and muscle strength than non-WC subjects [52]. Moreover, in this study, the risk of developing sarcopenia, and in particular sarcopenic obesity was nearly six times higher in participants with severe WC compared with participants at stable weight, independently from age and other confounding factors [52].

In a large group of elderly men and women from the Health ABC Study, participants with involuntary weight loss and regain during the 6-year follow-up, despite having similar amounts of lean and fat mass compared to weight-stable individuals during the study, developed poorer physical function and increased risk of mobility disability in a following period of 8 years of study [55]. Moreover, involuntary weight loss and regain was associated with increased risk of mortality and hospitalization, also after multiple adjustments [55]. However, these results are difficult to be interpreted since in this study a high proportion (until 55%) of participants reported that they were not trying to lose or gain weight, which suggests that the presence of unintentional weight loss may exert different effects on body composition and on health-related outcomes during weight changes [55].

General limitations of these studies are represented by their retrospective analyses, since weight changes were reported by patients, and finally the lack of information about the type and amount of physical activity performed [52, 55]. Moreover, the existence of different definitions of sarcopenia as well as of weight loss adds even more complexity to this yet unexplored area of research.

## Weight cycling and body composition: possible confounders

**Fig. 1:**
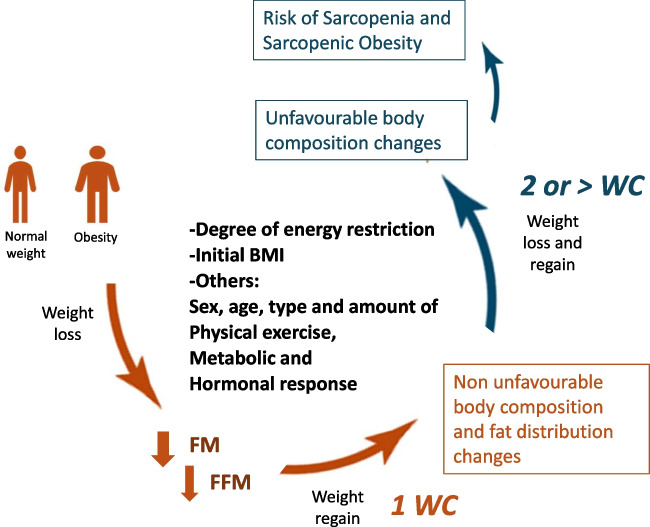
Weight cycling (WC) and body composition changes as possible determinants of Sarcopenia and Sarcopenic Obesity: analysis of possible counfounding factors

Several factors should be taken into account in the complex interplay between WC, body composition changes and the risk of sarcopenia (Fig. 1). Methods for body composition evaluation, type of nutritional intervention, interference of physical exercise as well as age, BMI and other characteristics of enrolled subjects, are all factors that could influence the results and prevent their generalization to larger populations.

More importantly, there is no consensus on a unique definition for WC [7, 8]. The literature proposes different definitions of WC, depending firstly on the number of cycles of weight loss and regain, secondly on the magnitude and on the timing of weight loss and regain, and thirdly on the variable used to measure WC (weight in kilograms or percentage of weight) [7, 8].

### Body composition methods

The majority of the studies used underwater weighing [31, 32, 34, 36, 38], bioimpedance analysis (BIA) [33, 41, 44, 50, 51, 53, 56] or Dual Energy X-ray Absorptiometry (DXA) [35, 40, 42, 43, 45–49, 52, 54, 55], whereas only a few employed Computed tomography (CT) [37, 49] or Magnetic Resonance Imaging (MRI) [35, 46] for reasons of cost and applicability of these methods to large populations. The precision of these methods differs and thus their capacity to detect true changes in body compartments after weight changes [67–69].

Gold standard methods for body composition such as CT or MRI (i.e. more precise methods able to recognize even small changes) were used in studies evaluating a single weight cycle and were mostly focused on fat distribution changes [35, 37, 46]. The only study that also evaluated muscle thigh by using CT in subjects aged 65–79 years with overweight and after WC was that of Chmelo et al. [49], and it reported a significant loss of thigh muscle volume.

Eight studies using underwater weighing, BIA or DXA to evaluate body composition changes after WC reported no significant changes in FM and/or FFM [33–38, 46, 51], 3 observed only FM increase [32, 41, 43], 5 only FFM loss [39, 40, 47, 49, 52], and the remaining both FM increase and FFM loss [42, 44, 50, 53] (Table 1). However, the discrepant results observed in these studies may depend by the fact that underwater weighing, BIA and DXA show minimum detectable change in both compartments ranging between 1 to 2 kg, and even higher if used in subjects with obesity [67]. In these studies, the fact that body composition changes are small and close to the limit of precision of each method makes difficult to interpret their results [67].

### Interference of physical exercise

Physical exercise, mostly resistance training, is known to preserve FFM loss observed after dieting and losing weight [70, 71]. In the majority of papers, no specific advice was given to subjects with weight problems involved in nutritional programs and no information is available about the basal degree of physical activity of enrolled subjects that should be taken into account in the analysis of body composition changes as a potential confounder [31–37, 39–41, 43, 46, 52, 53] (Table 1).

An intervention with a program of physical exercise besides diet was present in only 10 out of 23 studies that examined the relation between WC and body composition [31, 38, 42, 44, 45, 47–51]. In two studies [42, 49] where resistance exercise was associated with diet, as well as in others [31, 44] with both strength and aerobic exercise associated with diet, FFM loss was not preserved by the intervention. Similarly, FFM loss was observed, with or without FM increase, in all the other studies with aerobic exercise [38, 45, 47, 48, 50, 51]. However, these results are difficult to interpret because information about exercise compliance is not available and the level of exercise besides dieting period is not known.

### Number of weight cycles and length of follow-up

The definitions of WC differ from each other for the magnitude and the timing of weight loss and regain, but mostly for the number of weight cycles considered [31–53].

Eleven out of 23 studies examined body composition changes after only one WC [35–38, 40, 45–49, 51] and the others after two or more [31–34, 39, 41–44, 50, 52, 53] (Table 1). After 2 or more WC the majority of the studies (8 out of 12) reported FM increase [41, 43] evaluated alone or associated [42, 44, 50, 53] or not [32, 34] with concomitant FFM decline, when contemporary measured. Whilst, after only one WC, most studies (6 out 12) did not observe any significant body composition change [35–38, 46, 51].

Thus, the number of WC seems to be relevant and suggests that the higher the number of WC, the greater the possibility of observing unfavorable body composition changes, especially in older adults.

Not only is the number of WC dissimilar in all the studies but the definition of WC is also different. Most studies used reported data about weight history to define WC [31–33, 42, 44, 52, 53], while only a few used the Weight and Lifestyle Questionnaire to get standardized data [34, 41, 43]. Moreover, the magnitude of the weight loss and regain also differ considerably from the studies included in this review: some authors defined WC by loss and regain of 5 or 10 kg [31, 33, 36, 40, 42, 52, 53] or by using a specific percentage of body weight [35, 44, 46, 47, 51] whereas others considered the number of diets [32, 42, 50] (Table 1).

Finally, even the follow-up period differed among studies; some that considered more than 1 weight cycle had longer follow-up.

### Age of the study population

In the majority of the studies, participants were young or middle-aged men and women [31–40, 43, 44, 46, 48, 50–53], and only in 5 out of 23 [41, 42, 45, 47, 49] were subjects older than 65 years enrolled (Table 1). In these studies, significant changes in body composition after WC were observed, but aging was not considered as a confounding factor; thus, it is difficult to distinguish between WC and aging effects.

In fact, aging itself may modify body composition independently by weight change [72]. Only a few studies evaluated WC effects on body composition in older populations and were performed in subjects with both voluntary [41, 42, 45, 47, 49] and involuntary weight changes [54–56]. In a study including 459 men and women with obesity and a wide age range (19–65 years), WC was positively related to FM% and waist in both genders [41]; however, this association disappeared after adjustment for age and BMI [41]. However, among the 622 participants from the Walking Away from Type 2 Diabetes, patients with involuntary weight loss and regain lost FFM, even after adjustment for age and other confounders [56].

The correlations between WC, body composition changes and aging are truly complex, and other studies are needed to interpret these interrelationships definitively [41].

## Conclusions and future perspectives

Review of the literature supports these conclusions:

1. Epidemic obesity is associated with an increased number of subjects who are dieting.
2. A substantial proportion of dieters undergo WC defined as intentional weight loss followed by unintentional weight regain.
3. Not only patients affected by overweight and obesity, but also normal weight subjects may experience WC.
4. There is strong concern about the effects of WC on morbidity. Association between WC and eating disorders as well as with other metabolic and cardiovascular morbidity has been observed.
5. Effects of WC on body composition changes may be underestimated by a) low precision of methods used to estimate body composition, b) interference of physical exercise, c) number and definition of WC, d) age of the population.

Keeping in mind the above confounders it seems possible to conclude that:

- the more precise the method used to assess body composition changes, the higher the number of WC, the higher the age of study population, the more evident is the fact that, with WC, lower FFM is regained and thus a mismatch between muscle and fat happens;
- weight changes in old age, as shown in studies evaluating also involuntary weight loss and regain, leads to loss of FFM;
- evaluation of muscle strength and physical performance changes, besides body composition assessment, may be clinically relevant and help to clarify better whether WC may induce sarcopenia and sarcopenic obesity.

### Statements

MZ had the idea for the article, draft and critically revised the work, AG performed the literature search and articles analysis and drafted the manuscript; FF and APR performed the literature search and drafted the manuscript; GM drafted and revised the manuscript; EZ performed the literature search and data analysis, drafted and critically revised the work.

## Data Availability

No datasets were generated or analysed during the current study.

## Abbreviations

WC: Weight cycling
FFM: Fat free mass
LBM: Lean body mass
FM: Fat mass
FM%: Fat mass percentage
VLCD: Very low-calorie diet
CT scan: Computed Tomography scan
WHR: Waist to hip ratio
VAT: Visceral adipose tissue
ASM: Appendicular skeletal muscle mass
BIA: Bioimpedance analysis
DXA: Dual Energy X-ray Absorptiometry
CV: Coefficient of variation

## Declarations

Informed consent statement: not applicable.
